# Emotional Regulation Following Acquired Brain Injury: Associations With Executive Functioning in Daily Life and Symptoms of Anxiety and Depression

**DOI:** 10.3389/fneur.2020.01011

**Published:** 2020-09-10

**Authors:** Jan Stubberud, Marianne Løvstad, Anne-Kristin Solbakk, Anne-Kristine Schanke, Sveinung Tornås

**Affiliations:** ^1^Department of Psychology, University of Oslo, Oslo, Norway; ^2^Department of Research, Lovisenberg Diaconal Hospital, Oslo, Norway; ^3^Department of Research, Sunnaas Rehabilitation Hospital, Nesodden, Norway; ^4^RITMO Centre for Interdisciplinary Studies in Rhythm, Time and Motion, University of Oslo, Oslo, Norway; ^5^Department of Neurosurgery, Oslo University Hospital, Olso, Norway; ^6^Department of Neuropsychology, Helgeland Hospital, Mosjøen, Norway

**Keywords:** brain injury, emotional regulation, executive function, psychological distress, assessment

## Abstract

**Objective:** To examine whether a questionnaire measuring emotional regulation after acquired brain injury adds clinical information beyond what can be obtained with a comprehensive executive function questionnaire and an anxiety and depression measure.

**Method:** Seventy adult persons (age 19–66 years, *M*_age_ = 43, *SD*_age_ = 13) with acquired brain injury in the chronic phase and executive function complaints. All were recruited to participate in a randomized controlled trial (NCT02692352) evaluating the effects of cognitive rehabilitation. Traumatic brain injury was the dominant cause of injury (64%), and mean time since injury was 8 years. Emotional regulation was assessed with the Brain Injury Trust Regulation of Emotions Questionnaire (BREQ). Executive function was assessed with the Behavior Rating Inventory of Executive Function Adult Version (BRIEF-A). The Hopkins Symptom Checklist 25 (HCSL-25) was employed to measure anxiety and depression symptoms.

**Results:** Overall, significant correlations were found between reports of emotional regulation (BREQ) and executive function in daily life (BRIEF-A). Furthermore, our analyses revealed a significant relationship between self-reported scores of emotional regulation (BREQ) and symptoms of anxiety and depression (HSCL-25).

**Conclusion:** The significant associations between the BREQ and most of the other clinical measures indicate that, for patients with acquired brain injury, the BREQ does not add substantial information beyond what can be assessed with the BRIEF-A and the HSCL-25.

## Introduction

Difficulties in emotional regulation are among the most common and debilitating consequences of acquired brain injury (ABI), such as traumatic brain injury (TBI) and cerebrovascular accidents (CVA), with potential deleterious effects in all life domains [e.g., (1–4)]. Indeed, impaired emotional regulation can lead to compromised social functioning, decreased leisure activity, increased risk of suicide, and loss of employment/failure to return to work (3, 5–7). Despite its clinical significance, relatively little research has systematically addressed emotional regulation in individuals with ABI. Likely contributing to the lack of research in this area is the absence of instruments that adequately assess the complexity of this construct among adults with ABI. Accurate evaluation of the nature of deficits in emotional regulation is, however, imperative in the process of developing suitable and realistic rehabilitation and therapeutic intervention plans after ABI.

Emotional regulation relates to the capacity to flexibly modulate and control subjective experience and expression of emotions (8, 9), and the reduction of emotional arousal (10). In ABI, there may be impairments in self-monitoring and control, in addition to the ability to differentiate emotions, that are revealed through various symptoms of emotional dysregulation, including disinhibited emotion/behavior, and reduced emotional awareness and expression (8, 11). Further, emotional regulation is an important aspect of executive functioning (EF) (12, 13), broadly described as inter-related top-down processes promoting the control and regulation of cognition, behavior, and emotion (14). In contrast to the view that brain injury is directly responsible for emotional dysregulation, it can also represent secondary reactions to the consequences of ABI (15). Importantly, the experience of cognitive deficits after ABI has been described as having a “disordered” mind (16), a situation that can be emotionally experienced as a disorganized inner state. Adding layers of complexity, pre- and comorbid emotional problems may also influence symptom presentation after ABI. Nevertheless, the reactions to the psychosocial and cognitive changes associated with having an ABI makes it challenging to conceptualize and measure problems with emotional regulation (17).

Most measures addressing emotional functioning were not specifically developed for ABI and often focus on the phenomenology of depressive or anxiety states, rather than the actual capacity to regulate emotion. Thus, Cattran et al. (8) developed a questionnaire to measure emotional regulation after ABI, the Brain Injury Rehabilitation Trust Regulation of Emotions Questionnaire (BREQ). To our knowledge, only two feasibility studies (18, 19), the original study by Cattran et al. (8) and a cognitive rehabilitation study (20), have employed the BREQ in the field of ABI. Also, the original study by Cattran et al. is the only one providing BREQ data from relatives of ABI individuals (i.e., informants) (8). Hence, there are a modest number of empirical studies involving the BREQ. In addition, no studies have examined its relationship with a comprehensive EF questionnaire, such as the Behavior Rating Inventory of Executive Function Adult Version (BRIEF-A; 21). Cattran et al. (8), however, examined the association of BREQ with the Dysexecutive Questionnaire [DEX; (21)] and demonstrated strong correlations. Still, the DEX only contains 20 items, and few of these address emotional functioning. In summary, the multitude of problems related to reduced emotional regulation after ABI, along with the lack of relevant measurement tools that are necessary when differential diagnoses are considered, highlight the importance of generating more knowledge about the clinical properties of the BREQ (emotional regulation), including establishing its association to various EF domains and symptoms of anxiety and depression.

The present article reports on a subset of baseline data from Tornås et al.'s (20) randomized controlled trial (RCT; *n* = 70), where the efficacy of Goal Management Training (GMT) was examined in patients with ABI.

The main goal of the current study was to examine the relationship between the BREQ and (a) a questionnaire measure of EF in daily life (BRIEF-A) and (b) symptoms of anxiety and depression, as measured by the Hopkins Symptom Checklist 25 [HSCL-25; (22)], in persons with ABI in the chronic phase. Based on the sparse extant literature, it was expected that:

1. Both self- and informant reports of BREQ and BRIEF-A [i.e., Global Executive Composite (GEC), Behavioral Regulation Index (BRI), and Emotional Control subscale] would be significantly correlated (hypothesis 1).
2. Intercorrelations between BREQ and HSCL-25 would occur (hypothesis 2).

## Method

This study reports baseline data from a large single-center RCT (20). All participants provided written informed consent. The study was approved by the Regional Committee for Medical Research Ethics (2012/1436, South-Eastern Norway) and conducted in accordance with the Helsinki Declaration. Clinical Trial Registration No.: NCT02692352.

### Participants

An information letter was sent to 178 former patients (aged 18–67 years) at Sunnaas Rehabilitation Hospital (SRH) with a verified ABI and self-reported executive difficulties in daily life, at least 6 months post-injury. Any neurodegenerative disorder, ongoing substance abuse, major psychiatric diseases, and/or severe cognitive deficits were exclusion criteria.

Informed consent was returned from 90 persons who underwent a comprehensive screening interview by phone. Fourteen declined participation and 6 did not meet inclusion criteria. Thus, the final sample totaled *n* = 70 (age 19–66 years, *M*_age_ = 42.9, *SD*_age_ = 13), with 69 participants returning the questionnaires used in the present study. Fifty-eight participants (83%) had previously received subacute rehabilitation at SRH. Traumatic brain injury was the dominant cause of injury (64.3%), and a slight majority were males (52.9%). All participants were Caucasian. Mean time since injury was 8 years (*SD* = 112.4 months), ranging from 10 to 575 months. The mean length of education was 13.4 years (*SD* = 2.4) (Tables 1, 2). About one third (32.9%) of the sample received disability pension, and the rest were either in vocational rehabilitation, working (part- or full-time), students, or on sick leave. All participants were independent in ADL.

**Table 1:** Demographic, cognitive, and brain injury characteristics of the participants.

	**Total (*n* = 70)**
Age, mean ± SD	42.89 (12.96)
Gender, *M* = men	38 M (54.3)
Education, years ± SD	13.4 (2.43)
WASI	104.31 (12.65)
Tower Test	10.37 (2.82)
Months since injury ± SD	97.47 (112.44)
Injury etiology *n* (%)	
TBI	45 (64.3)
Stroke	15 (21.5)
Tumor	6 (8.6)
Anoxic	2 (2.9)
Other	2 (2.9)
Vocational status *n* (%)	
Work (full time)	6 (8.8)
Work (part time)	7 (10)
Voc. Rehab	25 (35.7)
Sick leave	3 (4.3)
Student	6 (8.6)
Disability pension	23 (32.9)
In a relationship *n* (%)	44 (63)

*Percentage totals may not add to 100% due to rounding. Sign, Significance; Voc. rehab, Vocational rehabilitation; WASI, Wechsler Abbreviated Scale of Intelligence. Scaled scores on the WASI and Tower Test*.

**Table 2:** Radiological description of the brain injuries.

CT/MRI verified ABI at onset *n* (%)	
Yes	36 (97.3)
No^a^	1 (2.7)
MRI verified lesion at baseline *n* (%)	
Yes^b^	23 (62.2)
No	8 (21.6)
Missing^c^	6 (16.2)
Injury localization *n* (%)	
Right hemisphere	7 (18.9)
Left hemisphere	9 (24.3)
Bilateral	5 (13.5)
Frontal	11 (29.7)
Parietal	4 (10.8)
Temporal	7 (18.9)
Occipital	0
Cerebellum	2 (5.4)
Subcortical nuclei^d^	1 (2.7)
Subcortical white matter	13 (35.1)
Cortical atrophy *n* (%)	35 (50)

a *Verified by neurological and neuropsychological evaluation*.

b *MR/CT scans were collected from other hospitals for five participants due to practical or medical reasons; the images were interpreted by the same radiologist. All 5 scans were performed between 2011 and 2013*.

c *MRI was not possible to conduct due to practical reasons for four participants, medical reasons for four, and one participant refused to undergo repeated scanning*.

d *Striatum, basal ganglia and/or thalamus. MRI, Magnetic Resonance Imaging*.

For 56 of the participants, magnetic resonance imaging (MRI) was obtained in the study period using a 3 Tesla scanner (Achieva 3.0T, Philips Medical System, Best, The Netherlands) at the Intervention Center at Oslo University Hospital. Previous MRI/computed tomography scans were collected from other hospitals for five participants. For various reasons, scanning could not be performed for nine participants. The frontal lobe was the most affected cortical location, followed by temporal- and parietal lobe injury. Finally, about 50% of the sample had signs of cortical atrophy (Table 2).

All participants were asked to give consent for the two questionnaires to be sent to an informant that knew them very well. Two of the participants declined, and four informants did not return the questionnaires. Therefore, 64 informants (all Caucasian) were included, with 57 and 56 informants completing the BREQ and the BRIEF-A, respectively. More than half of the informants (53.1%) were spouses/partners living with the participants, about one third (31.3%) were parents, and the remaining informants were siblings (12.5%), adult children (3.1%), or close friends (7.8%).

### Measures

In the main study (20), various neuropsychological tests and questionnaires were administered to the participants. For the current supplementary study, the Wechsler Abbreviated Scale of Intelligence [WASI; (23)] and the Tower Test (24) were included to characterize the cognitive functioning [i.e., general intellectual capacity (IQ) and EF] in the sample. Daily life EF was assessed with the BRIEF-A (25), emotional regulation was measured with the BREQ (8), and symptoms of anxiety and depression were assessed with the HSCL-25 (22).

#### The Brain Injury Rehabilitation Trust Regulation of Emotions Questionnaire

The BREQ is a 32-item standardized questionnaire that aims at assessing changes and disturbances in emotional regulation following ABI, yielding a total sum score. Patients and a significant other are asked to rate each item's frequency of occurrence on a 4-point Likert scale from 1 (*never*) to 4 (*always*). The measure has demonstrated good concurrent validity (*r* = 0.64–0.82) and to be distinguishable from measures of cognitive ability and tests of affect (8).

#### The Behavior Rating Inventory of Executive Function Adult Version

The BRIEF-A is a 75-item standardized questionnaire of an adult's EF or self-regulation in his or her everyday environment. Based on 9 sub-scales, it yields a GEC score as well as two Composite Index scores: the BRI and the Metacognition Index (MCI). The Emotional Control scale measures the impact of EF problems on emotional expression and assesses the ability to modulate or control emotional responses. Patients and a significant other are asked to rate each item's frequency of occurrence on a 3-point Likert scale from 1 (*never*) to 3 (*often*) (25). The BRIEF-A's reliability is high; Cronbach's alpha of BRI and MI has been found to be 0.94 and 0.96, respectively (26). The BRIEF-A informant form was administered to the same significant other informant as the BREQ.

#### Hopkins Symptom Checklist 25 (HSCL-25)

The HSCL-25 is a 25-point self-report inventory of depressive and anxiety symptoms (22). It includes a 15-item depressive symptoms scale and a 10-item anxiety symptoms scale. Items are scored on a Likert scale ranging from 0 (*not at all*) to 4 (*very much*). Finally, the HSCL-25 has satisfactory validity and reliability as an instrument of anxiety and depression symptoms (22).

### Statistical Analyses

Data analyses were conducted using SPSS version 25.0 for Windows. Frequency distributions, means, and standard deviations (*SD*) were calculated for the demographic, medical, and cognitive variables. Relationships between measures were examined with Pearson product-moment correlation coefficients (two-tailed test). For BRIEF-A, the GEC, BRI, MCI, and Emotional Control subscale in both self- and informant reports were selected as variables, while the total scores were employed for BREQ and HSCL-25. In determining the strength of the relationships, Cohen's (27) guidelines were employed: *r* = 0.10–0.29 (small), *r* = 0.30–0.49 (medium), and *r* > 0.50 (large). A conservative alpha-level of 0.01 was applied in order to take multiple comparisons into account.

## Results

### Descriptive Data on General Intellectual Capacity (IQ), Executive Functioning, and Questionnaires

The ABI group had general intellectual capacity (IQ) and EF test performance within the normal range, relative to the standardization samples (Table 1). The BREQ-self mean score was 55.7 (*SD* = 14.4), while the BREQ-informant mean score was 52.5 (*SD* = 13.1). To our knowledge, there are no recommendations available regarding a clinical cut-off score on the BREQ, nor any published data from healthy controls. However, with the rating “always” (4), “often” (3), “sometimes” (2), or “never” (1), we decided to use an item mean of ≥ 2.5 with total score ≥ 80 as cut-off score. Hence, when adding up how many got a total score ≥ 80, 10% (*n* = 7) self-reported ≥ 80, and 3% (*n* = 2) of the informants reported a score ≥ 80 in the patients. Moreover, two of the BRIEF-A (group average) scores were equal to or above recommended clinical cut-off (≥ *T* = 65), i.e., GEC-self (*M* = 64.7, *SD* = 9.7) and MCI-self (*M* = 65.3, *SD* = 9.8). On an individual level, a score equal to or above recommended clinical cut-off was self-reported for 52% (*n* = 36) on the GEC, 38% on the BRI (*n* = 26), 54% (*n* = 37) on the MCI, and 41% (*n* = 28) on the Emotional Control scale. For the informants, a clinical score was reported for 25% (*n* = 14) on the GEC, 18% (*n* = 10) on the BRI, 38% (*n* = 21) on the MCI, and 13% (*n* = 9) on the Emotional Control scale. However, all BRIEF-A-informant group scores were below cut-off (*T* ≤ 65). While the group average on the HSCL-25 (*M* = 22.4, *SD* = 15.3) was below the recommended clinical cut-off (HSCL-25 total < 25), 35% (*n* =24) reported symptoms of anxiety and depression above clinical cut-off (28).

### Relations Between BREQ and Brief-A (Self- and Informant Reports)

All correlations between BREQ-self and BRIEF-A (GEC and BRI) scores were large and positive, including GEC-self, *r* (67) = 0.55, *p* < 0.001, BRI-self, *r* (67) = 0.77, *p* < 0.001, Emotional Control-self *r* (67) = 0.74, *p* < 0.001, in addition to BRI-informant, *r* (54) = 0.51, *p* < 0.001 and Emotional Control-informant, *r* (54) = 0.54, *p* < 0.001 (Table 3). Additionally, all correlations between BREQ-informant and BRIEF-A (GEC and BRI) scores were positive, including GEC-self, *r* (55) = 0.44, *p* < 0.001 (medium), BRI-self, *r* (55) = 0.46, *p* < 0.001 (medium), Emotional Control-self, *r* (55) = 0.48, *p* < 0.001 (medium), GEC-informant, *r* (54) = 0.71, *p* < 0.001 (large), BRI-informant, *r* (54) = 0.84, *p* < 0.001 (large), and Emotional Control-informant, *r* (54) = 0.81, *p* < 0.001 (large) (Table 3). Finally, for the MCI only the informant reports of BREQ and BRIEF-A reached significance, *r* (54) = 0.55, *p* < 0.001 (large) (Table 3).

**Table 3:** Correlations between the BREQ and the BRIEF-A and HSCL-25.

	**BREQ-S**	**BREQ-I**
BRIEF-S-GEC	0.55**	0.44**
BRIEF-S-BRI	0.77**	0.46**
BRIEF-S-MCI	0.21	0.32
BRIEF-I-GEC	0.30	0.71**
BRIEF-I-BRI	0.51**	0.84**
BRIEF-I-MCI	0.12	0.55**
BRIEF-S-EMO	0.74**	0.48**
BRIEF-I-EMO	0.54**	0.81**
HSCL-25 Total	0.52**	0.29

*BREQ, BIRT (Brain Injury Rehabilitation Trust) Regulation of Emotions Questionnaire; BREQ-S, self-rated version of the BREQ; BREQ-I, informant-rated version of the BREQ; HSCL-25, Hopkins Symptom Checklist 25; BRIEF-A, Behavior Rating Inventory of Executive Function Adult Version; BRIEF-S/I, BRIEF self/informant version; EMO, Emotional control; GEC, Global Executive Composite; BRI, Behavioral Regulation Index; MCI, Metacognition Index*.

** *Correlation is significant at the 0.001 level (two-tailed)*.

### Relations Between BREQ (Self- and Informant Reports) and HSCL-25

There was a positive significant correlation between BREQ-self scores and anxiety and depression scores (HSCL-25), *r* (61) = 0.52, *p* < 0.001 (large). However, the correlation between BREQ-informant and patient-rated HSCL-25 scores did not reach statistical significance, *r* (49) = 0.29, *p* = 0.03 (Table 3).

### Post-hoc Analyses

In order to allow comparison of results, one sample *t*-tests were conducted to determine if there were significant differences between the BREQ reports in our study and the BREQ reports from Cattran et al.'s study (age 18–61 years, *M* = 36, *SD* = 12) (8). There was no difference between the mean BREQ-self score from our ABI sample (*M* = 55.7, *SD* = 14.4) and the mean BREQ-self score from the ABI cohort studied by Cattran et al. (*n* = 72, *M* = 58.23, *SD* = 20.01), *t* (68) = 1.31, *p* = 0.196. However, the mean BREQ-informant score from our sample (*M* = 52.5, *SD* = 13.1) was significantly lower than the mean BREQ-informant score from Cattran et al.'s study (*M* = 63.26, *SD* = 19.54), *t* (56) = 6, *p* < 0.001.

## Discussion

The main aim of the present study was to examine the relationship between self- and informant perceived emotional regulation and daily life EF, and emotional regulation and symptoms of anxiety and depression, in persons with ABI. Overall, several findings supported our hypotheses.

### Reported Emotional Regulation and Executive Function in Daily Life

Consistent with the first hypothesis, both self- and informant reports of perceived emotional regulation and daily life EF were significantly correlated. For both BREQ versions, the strongest associations were observed with the BRI in the BRIEF-A. As this index is composed of scales designed to measure the ability to maintain appropriate regulatory control of behavior and emotional responses (i.e., Inhibit, Shift, Emotional Control, and Self-Monitor), it is more closely related to the domain of emotional regulation than the BRIEF-A MCI (8, 11, 25). Of note, an association between the BRIEF-A Emotional Control subscale and the BREQ was also found. A relationship between BREQ and MCI informant-report was also detected. The division between the BRI and MCI is mainly theoretical, and some of the abilities reflected in the MCI (i.e., Initiate, Working Memory, Plan/Organize, Task Monitor, Organization of Materials) may also overlap with aspects of emotional regulation. Clearly, the constructs of emotional regulation and EF are closely connected (13), as the measures are intended to assess everyday manifestations of emotional dysregulation and executive dysfunction, respectively. The BRIEF-A includes behavioral and emotional aspects, such as appropriate inhibition of thoughts and actions, flexibility in shifting problem-solving set, modulation of emotional response, and monitoring of one's activities, that are very important for emotional regulation (8, 9). The findings in the present study are in accordance with previous research (8), showing a strong relationship between BREQ and measures of EF. It is possible that the BREQ represents a valuable contribution to the assessment of emotional regulation in the ABI-population, but it is still uncertain what additional information it adds beyond the BRIEF-A. Due to an often observed discrepancy between objective and subjective measures of cognition, with a generally poor relationship between questionnaires and performance-based neuropsychological tests (29), one might consider employing multiple EF measures in future studies. Furthermore, as there are no published studies that can inform on recommendations regarding a clinical cut-off score on the BREQ, nor any published BREQ data from healthy controls, it is difficult to know if our sample experienced emotional dysregulation in the clinical range, based on the BREQ. Nevertheless, only 10% of the participants self-reported, and 7% of the informants reported a total score at or above what we suggest as the clinical cutoff (≥80). This finding is somewhat in contrast to the Emotional Control (BRIEF-A) scores, where 41% self-reported and 13% of the informants reported scores in the clinical range. Although conjectural, our *post-hoc* analyses revealed that the informants in the present study reported significantly less problems with emotional regulation in the patients compared to the ABI sample in Cattran et al.'s study (8). The ABI-participants in the latter study (8) were, however, slightly younger and with a lower IQ relative to our sample. It is important to consider that several factors might contribute to bias, and differences, in patient and informant ratings (e.g., cognitive deficits, severe emotional regulation dysfunction, self-awareness, social desirability bias, informant's burden, abuse, stress level, and/or personality) (30–33), suggesting that information should be gathered from multiple sources. Finally, the majority of the participants chose a spouse/partner as an informant, and the remaining informants were parents, siblings, friends, or adult children. Due to the variability of the informants and their relationship with the patients, potential differences between spouse/partner reporting and the reporting of the other informants were examined. In our *post-hoc* analyses, no significant differences were, however, detected between spouse/partner reporting and the reporting of the other informants.

### Perceived Emotional Regulation and Symptoms of Anxiety and Depression

With regard to the second hypothesis, a statistically significant relationship between scores of emotional regulation (self-reported) and symptoms of anxiety and depression was detected. Although emotional regulation has been given relatively little attention in the field of ABI, it is among the most studied phenomena in contemporary psychology, having generated a robust body of evidence linking it to psychopathology (34), in addition to being recognized as a core function supporting psychological well-being (35). In our study, both the self- and informant-rated BREQ versions produced moderate to high correlations with the HSCL-25, which may suggest either that the BREQ also measures a degree of anxiety and depression, or that psychological distress is prevalent among those suffering from emotional dysregulation. These findings are, however, in accordance with Cattran et al. (8) where moderate to high correlations between the BREQ and the Anxiety subscale of the Irritability, Depression, and Anxiety Scale were observed.

### Study Limitations

Clearly, studies with larger sample sizes are needed to more definitively examine the relationships between the questionnaires. The current sample was likely not representative of the entire population of individuals with ABI, as the RCT included participants who self-reported executive deficits, and were motivated for a cognitive rehabilitation intervention for dysexecutive symptoms. Thus, they potentially represent a group with high symptom awareness. In this regard, symptom validity measures, in addition to awareness questionnaires (self- and informant reports), should be considered for future studies. Importantly, since we included participants ranging from 10 months to 48 years post-injury, they were at different stages in their recovery processes, and thus had different functional status. Further, as about one third of the sample was on a disability pension, a majority in a relationship, and only 7% of informants reported emotion regulation dysfunction, this likely reflects a less severely injured segment of the ABI population (36, 37). Finally, a more detailed description of potentially complicating premorbid or comorbid factors, information about treatments received in the acute or subacute period, and symptom debut is recommended for future studies.

## Conclusion

This is the first study to examine the relationship between BREQ and a comprehensive EF questionnaire. Reports of emotional regulation and perceived EF in daily life were found to correlate in a sample of ABI participants. Furthermore, a relationship between scores of emotional regulation and symptoms of anxiety and depression was also detected. These findings indicate that, for patients with ABI, the BREQ does not add substantial information beyond what can be assessed with the BRIEF-A and the HSCL-25. Considering the covariation between the measures, and the lack of published norms for the BREQ, a preliminary recommendation is that it is premature to employ the BREQ as a standard measure for assessing emotional regulation in ABI.

## Data Availability Statement

The raw data supporting the conclusions of this article will be made available by the authors, without undue reservation.

## Ethics Statement

The studies involving human participants were reviewed and approved by Regional Committee for Medical Research Ethics, South-Eastern Norway. The patients/participants provided their written informed consent to participate in this study.

## Author Contributions

JS, ML, A-KSo, A-KSc, and ST contributed to the design and implementation of the research and to the analysis of the results. JS wrote the paper with input from all authors. All authors contributed to the article and approved the submitted version.

## Conflict of Interest

The authors declare that the research was conducted in the absence of any commercial or financial relationships that could be construed as a potential conflict of interest.
